# Genetic Diversity of *Campylobacter concisus* Isolates from Slovenian Patients with Infectious Diarrhoea

**DOI:** 10.3390/microorganisms14010087

**Published:** 2025-12-31

**Authors:** Romina Kofol, Mateja Pirs, Tadeja Kotar, Tatjana Lejko Zupanc, Andraž Celar Šturm, Andreja Kukec, Tadeja Matos, Tina Triglav

**Affiliations:** 1 Institute of Microbiology and Immunology, Faculty of Medicine, University of Ljubljana, 1000 Ljubljana, Slovenia; romina.kofol@mf.uni-lj.si (R.K.); mateja.pirs@mf.uni-lj.si (M.P.); andraz.celar-sturm@mf.uni-lj.si (A.C.Š.); tadeja.matos@mf.uni-lj.si (T.M.); 2 Division of Infectious Diseases, University Medical Centre Ljubljana, 1000 Ljubljana, Slovenia; tadeja.kotar@kclj.si (T.K.); tatjana.lejko@kclj.si (T.L.Z.); 3 Department of Public Health, Faculty of Medicine, University of Ljubljana, 1000 Ljubljana, Slovenia; andreja.kukec@mf.uni-lj.si

**Keywords:** *Campylobacter concisus*, WGS, virulence factors

## Abstract

*Campylobacter concisus* is recognized as a potential pathogen in gastrointestinal diseases, particularly in patients with chronic intestinal diseases. This study investigates the genomic characteristics, phylogenetic distribution, virulence factors, resistance genes and presence of plasmids in *C. concisus* isolates from Slovenian patients with community-acquired infectious diarrhoea. Prospectively collected isolates were analysed using whole-genome sequencing (WGS). WGS analysis revealed substantial genetic diversity among isolates, with distinct differences observed between two genomospecies (GS1, GS2). GS1 isolates had smaller genomes, lower GC content, and fewer coding regions than GS2 isolates. Multilocus sequence typing confirmed a high degree of genetic diversity, with most isolates belonging to novel sequence types. Plasmids, including pSma1 and pICON, were more prevalent in GS2 isolates. The virulence factors Zot and Exo9 toxins were detected in both genomospecies, with Zot predominantly found in GS1 and Exo9 in GS2. The T6 secretion system was prevalent in both groups, whereas the T4SS was less frequently observed. The combination of the T6SS, plasmids, and toxins suggests a complex mechanism of pathogenicity. This study highlights the high genetic diversity of *C. concisus* and provides new insights into its genomic features and virulence factors. The presence of plasmids and secretion systems, particularly the T6SS, underscores the potential of *C. concisus* for adaptation and pathogenicity.

## 1. Introduction

Infections caused by *Campylobacter* species are the most frequently reported zoonoses in the European Union (EU) [1]. Campylobacteriosis was the most frequently reported bacterial intestinal infection in Slovenia, with 38.6 reports per 100,000 inhabitants in 2023 [2].

Human infection typically occurs through the consumption of undercooked contaminated meat (especially poultry), unpasteurized milk, or contaminated water. Symptoms usually include watery diarrhoea, and in severe cases, bloody diarrhoea. Most infections resolve with fluid and electrolyte replacement, although antibiotic treatment may be necessary in severe cases. The most commonly used antibiotics are macrolides because high fluoroquinolone resistance often precludes empiric ciprofloxacin use in many regions of the EU [3]. The species most often responsible for human infections are *Campylobacter jejuni* and *Campylobacter coli*, although *Campylobacter upsaliensis* and *Campylobacter lari* are also occasionally implicated. In recent years, other *Campylobacter* species, including *C. concisus*, whose pathogenic potential is not fully understood, have been increasingly identified, partly due to improvements in molecular detection or changes in culture conditions [4,5,6].

*C. concisus* is part of the oral microbiota in many people, making it challenging to define its pathogenic role. The oral cavity serves as its likely natural reservoir, harbouring both virulent strains associated with infectious diarrhoea and non-pathogenic variants [7,8,9]. Colonization of the digestive tract likely occurs when the bacteria are introduced through the oral cavity and swallowed with saliva. Environmental factors such as pH changes and bile presence, as well as the individual’s immune status, influence colonization. Colonization with *C. concisus* has been observed in animals, such as poultry and pets. Poultry appears to be an important reservoir [8,10].

Understanding the pathogenic potential of *C. concisus* has progressed slowly due to its demanding growth conditions. Initially studied for its role in periodontal disease, recent research has focused on its involvement in intestinal diseases, including infectious diarrhoea and its potential role in chronic inflammatory bowel diseases. Some studies have associated *C. concisus* with gastroesophageal reflux disease and Barrett’s oesophagus, which may progress to oesophageal carcinoma [11,12,13]. Two distinct pathotypes of *C. concisus* have been described: the adherent-invasive *C. concisus* (AICC) and the adherent-toxigenic *C. concisus* (AToCC), which differ in their virulence mechanisms and are thought to play different roles in disease pathogenesis [14].

*C. concisus* is a genetically diverse species, with some strains being more pathogenic than others. It can adhere to and invade intestinal epithelial cells, causing inflammation and potential tissue damage. Proposed virulence factors for *Campylobacter* species include flagella-mediated motility, adhesion to intestinal mucosa, invasion of intestinal cells, secretion systems, and toxin production. However, the exact virulence mechanisms for *C. concisus* are still not fully understood [7,15,16,17,18].

Studies have shown genetic diversity among *C. concisus* [19,20] strains through multilocus sequence typing (MLST) and whole genome sequencing (WGS) [20,21,22,23]. WGS is a powerful tool for studying *C. concisus*, providing detailed genomic data that allow comprehensive analysis of virulence factors, resistance genes, genetic diversity, and plasmids that often carry genes related to antibiotic resistance and virulence. WGS can also be used to construct phylogenetic trees, revealing the evolutionary relationships between strains [24,25,26,27].

This study, the first of its kind in Slovenia, molecularly characterizes *C. concisus* isolates prospectively collected from patients with community-acquired diarrhoea, using WGS. By analysing the genetic diversity, virulence factors, and plasmid distribution in these strains, it provides a better understanding of the pathogenic potential of *C. concisus* and the factors that contribute to its ability to cause disease.

## 2. Materials and Methods

### 2.1. Sample Collection

This study included *C. concisus* isolates prospectively collected between 2016 and 2017 at the Institute of Microbiology and Immunology in collaboration with the Division of Infectious Diseases, University Medical Centre Ljubljana. Stool samples were obtained from adult patients with community acquired infectious diarrhoea (defined as three or more loose stools in 24 h lasting up to 14 days) and control group without diarrhoea [28]. During the prospective observational study, 388 stool samples (246 from adult patients with diarrhoea and 142 from control group) were included in the study protocol, which included standard cultivation in combination with membrane filtration and syndromic molecular testing for diarrheal pathogens. In 137 cases, we isolated at least one of the *Campylobacter* species, with *C. concisus* being identified in 59 of these cases (53 in patient group; 6 in control group). Because *C. concisus* is likely to contribute to the aetiology of gastroenteritis, especially in cases that have no known association with other established pathogens, the 53 isolates from patients were divided into two groups: Group A (N 24), in which only *C. concisus* was identified as a possible cause of community acquired infectious diarrhoea, Group B (N 29), in which *C. concisus* was identified together with another pathogen causing infectious diarrhoea (*C. jejuni*/*C.* *coli*, *Salmonella* spp., *Aeromonas* spp., *Shigella*/EIEC, *Clostridioides difficile*, *Cryptococcus* spp., *Giardia intestinalis*, norovirus, astrovirus, rotavirus A or adenovirus F40/41).

### 2.2. C. concisus Isolation and Identification

To isolate *C. concisus* from faeces, we used a cultivation method on a non-selective culture medium containing tryptose and sheep blood (Tryptic Soy Blood Agar, Oxoid, Wesel, Germany), following prior membrane filtration of the stool according to the modified Cape Town protocol [29,30,31]. For membrane filtration, polycarbonate membrane filters with a diameter of 47 mm and a pore size of 0.60 μm were used (Whatman Nuclepore, Sigma-Aldrich, MO, USA). The plates were incubated at 37 °C in a hydrogen-enriched microaerobic atmosphere for 72 h [31].

All suspicious colonies were identified at the species level using matrix-assisted laser desorption/ionization time-of-flight (MALDI-TOF) mass spectrometry on a Microflex Biotyper system (Bruker Daltonics, Bremen, Germany). The isolates were frozen and stored at −80 °C in a routinely used freezing mix containing Mueller–Hinton broth (Becton Dickinson BBL, Sparks, NV, USA) with 10% glycerol [32].

### 2.3. DNA Extraction

Genomic DNA for whole genome sequencing (WGS) was extracted using the DNeasy Blood & Tissue Kit (Qiagen Ltd., West Sussex, UK), following the manufacturer’s protocol for Gram-negative bacteria. The extracted DNA was stored at −80 °C until further use. DNA concentrations were measured using the Qubit 3.0 fluorometer in combination with the Qubit 1× dsDNA HS Assay Kit (both Thermo Fisher Scientific, Waltham, MA, USA). DNA purity was assessed based on the absorbance ratios at A260/280 and A260/230 using a NanoDrop 2000/2000c spectrophotometer (Thermo Scientific, Waltham, MA, USA). Only DNA samples with a concentration greater than 1 ng/µL and an A260/280 ratio between 1.8 and 2.0 were selected for WGS and included in further analysis.

### 2.4. Whole Genome Sequencing

A total of 54 isolates were sequenced using the NextSeq 2000 system (Illumina, San Diego, CA, USA) using 2 × 151 paired-end reads chemistry, aiming for a minimum coverage of 100×. Preparation of genomic DNA libraries was performed with the Illumina DNA Prep workflow (Illumina) according to the manufacturer’s instructions.

### 2.5. Bioinformatic Analysis

Acquired raw reads were filtered and trimmed using Fastp v0.23.2 [33]. The quality of both raw and trimmed reads was assessed with FastQC v0.11.9. Raw reads were assembled into contigs using SPAdes v3.15.5 with default parameters [34]. Quast v5.2.0 [35] and BUSCO v5.4.4 [36] were used for quality assessment of assembled genomes. The minimum threshold for a quality assembly was set at N50 score above 20,000, total number of contigs < 500, genome size within 10% of the reference genomes for each of the genomospecies, and BUSCO completeness of more than 95%. For further analyses, isolates were separated into two distinct genomospecies (GS1 and GS2) of *C. concisus* (16) based on GC content and genome size. FastANI v1.34 was used to perform pairwise average nucleotide identity (ANI) analysis on all the isolates to further confirm the distinction between genomospecies.

Assemblies were annotated using Prokka v1.14.6 [37], using the protein content of completed *C. concisus* genomes ATCC 33237 (GS1 (GCF_001298465.1)) and P1CDO3 (GS2 (GCF_003048685.2)) as references for genomospecies 1 and 2, respectively. Pangenome analysis for each of the genomospecies as well as all the isolates together, was performed using Roary v3.13.0 [38]. Analysis of gene homology was performed using zDB software v1.3.10 [39], which employs the orthogroup inference algorithm OrthoFinder [40] as well as Pfam domain analysis and COG (Clusters of Orthologous Genes) annotations.

### 2.6. MLST and Phylogenetic Analysis

**Figure 1:**
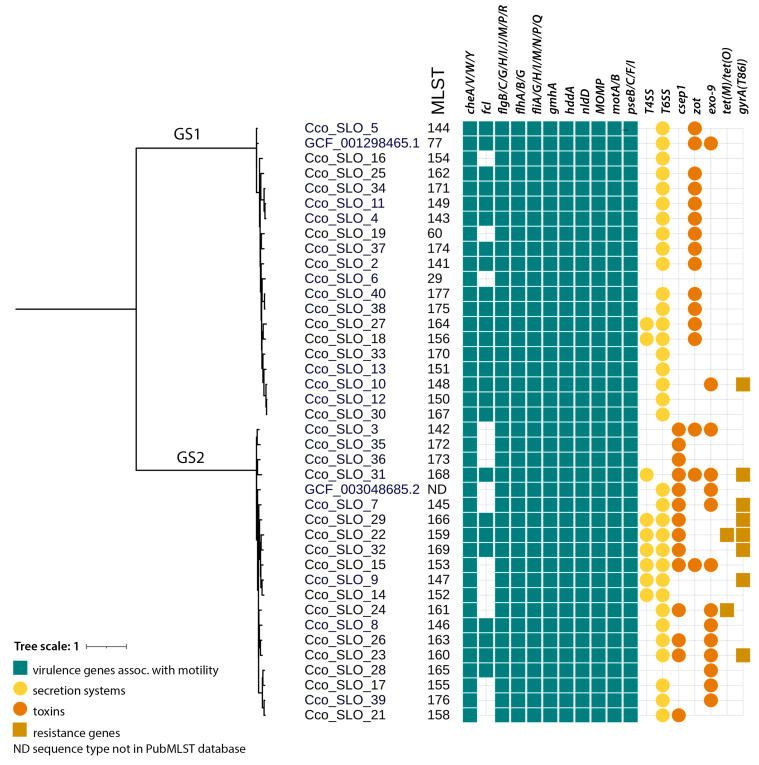
Phylogenetic tree with MLST, with distribution of *C. concisus* virulence genes (green rectangle), secretion systems (yellow circle), toxins (orange circle), and resistance genes (brown rectangle).

**Figure 2:**
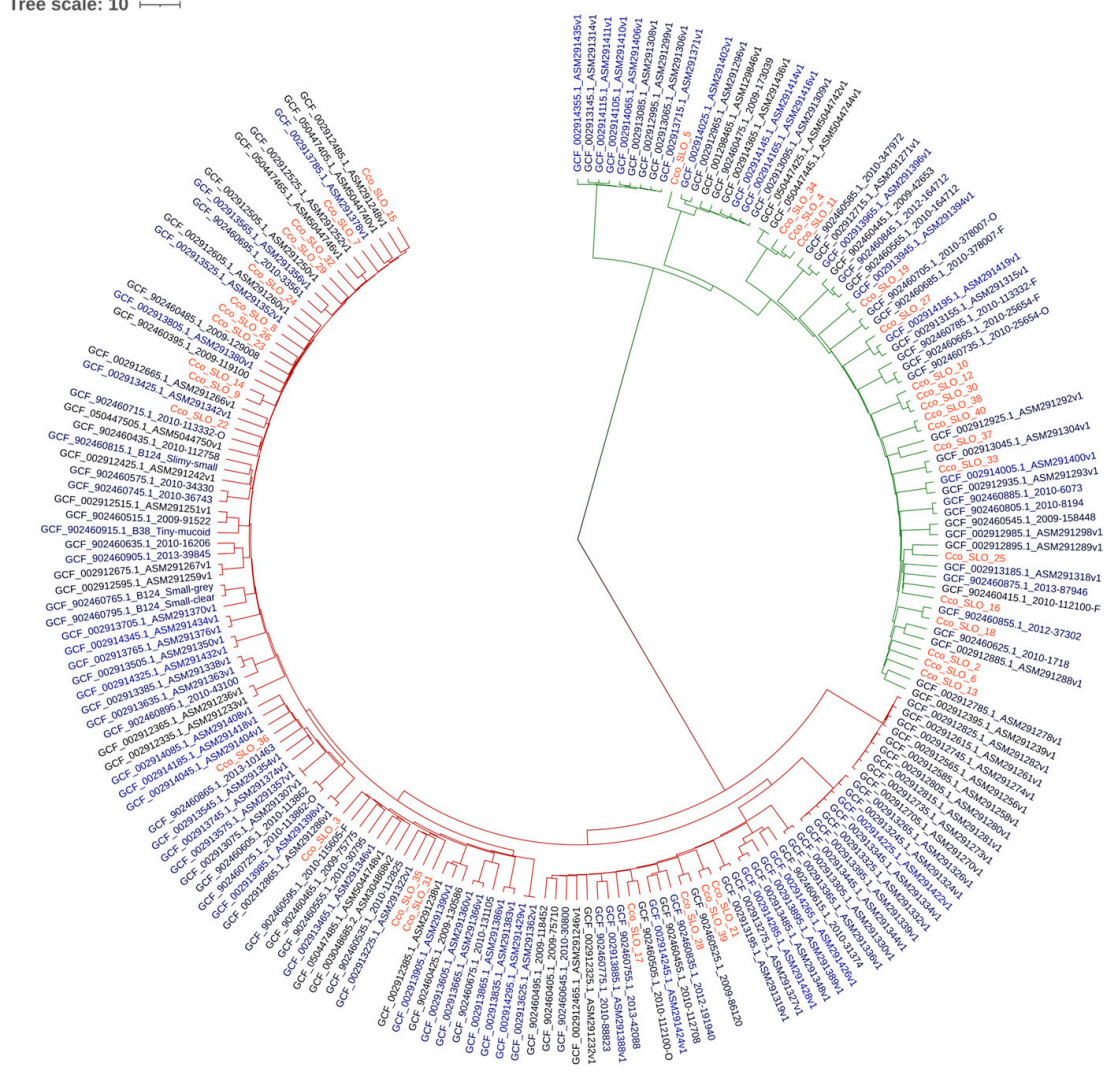
Phylogenetic tree of Slovenian *C. concisus* isolates and available European isolates. Dark red branches represent GS2, green branches represent GS1, and Slovenian isolates are marked in red.

Sequence typing was performed using PubMLST (17) with the *Campylobacter* non-*jejuni*/*coli* scheme (https://pubmlst.org/organisms/campylobacter-non-jejunicoli (accessed on 16 July 2024)). The *C. concisus/curvus* scheme is based on seven loci: *aspA*, *atpA*, *glnA*, *gltA*, *glyA*, *ilvD*, *pgm*. Two phylogenetic trees were constructed using ANIclustermap v2.0.1, based on average nucleotide identity, and visualized with iTOL [41]. The distinction between genomospecies within our samples is presented in Figure 1, while Figure 2 shows the phylogenetic similarity and distribution of Slovenian strains in comparison to European *C. concisus* genomes available in GenBank database.

### 2.7. Analysis of Antimicrobial Resistance, Virulence-Associated Genes and Plasmids

Genomic sequences were analysed with the CARD Resistance Gene Identifier [42] to determine the presence of acquired antimicrobial resistance genes and resistance-associated single-nucleotide polymorphisms.

A list of several virulence factors, previously mentioned in the literature for *C. concisus*, was created, along with select virulence-related genes of *C. jejuni*, listed in the Virulence Factor Database. Due to high genetic diversity among homologous genes in *C. concisus*, OrthoFinder was used to identify homologous gene clusters across all the genomes to determine the presence of chosen genes of interest. The presence of plasmids was assessed in two ways. First we used MOBsuite [43] to reconstruct and type plasmids; to avoid possible false negative results due to lacking databases for *C. concisus* plasmids, we checked the assembly graphs of each isolate with the Bandage visualization tool and looked for circular, closed contigs, not connected to the chromosomal contigs. Putative plasmid sequences were analysed with the blastn tool on the NCBI website, using the core_nt database. In cases in which there were no results with high reliability (coverage and identity scores above 90%), the plasmids were classified as novel.

Pathotypes were defined based on the presence of Exotoxin 9 (Exo9) and Zonula occludens toxin (Zot). Adherent-Invasive *C. concisus*, AICC is associated with chronic intestinal disease. In contrast, Adherent-Toxigenic *C. concisus* (AToCC) strains, which carry the *zot* gene, have been frequently isolated from patients with Inflammatory Bowel Disease (IBD) [44].

### 2.8. Statistical Analyses

To evaluate associations between the variables observed, we used the chi-squared (χ^2^) test. We applied the test under standard assumptions of independent observations and adequate expected cell counts. When assumptions were not met, we used Fisher’s exact test for small samples. Statistical significance was assessed using two-sided *p*-values with a threshold of *p* ≤ 0.05. All analyses were performed in IBM^®^ SPSS^®^ for Windows version 27 (SPSS Inc., IBM Company, Armonc, NY, USA) and Excel^®^ for Windows^®^ (Microsoft^TM^).

## 3. Results

In total, 59 isolates of *C. concisus* were obtained from stool samples and stored at –80 °C. We were only able to successfully revive 54 out of 59 isolates. Of the 54 draft genome assemblies, 40 passed all the quality checks and were used for further analysis; 16 from Group A and 22 from Group B. There were no statistically significant differences in patient age between the two groups. Patients in Group A reported blood in stool less frequently (0% vs. 18.2%, *p* = 0.007), had less vomiting (62.5% vs. 45.5%, *p* = 0.034), were more likely to be afebrile (56.3% vs. 27.3%, *p* = 0.014), and showed lower inflammatory markers with lower CRP levels (CRP > 52.0: 25% vs. 63.6%, *p* = 0.021) and a lower rate of leucocytosis (>7.75: 56.3% vs. 68.2%, *p* = 0.048), compared with Group B. There were no differences in stool frequency between the groups. In Group B, bacterial pathogens were the most commonly detected, including *Campylobacter jejuni* (20.7%), *Salmonella* spp. (20.7%), and *Shigella* spp. (6.9%), followed by viruses, primarily Norovirus (13.8%) and Rotavirus (13.8%). Parasites were identified in only one case (3.4% *Giardia intestinalis*).

The comparison of *C. concisus* isolates did not reveal significant differences between the two patient groups, except for the presence of Zot (Table 1). Isolates from Group A were predominantly assigned to GS2 (56.3% [9/16]), and those from group B to GS1 (54.5% [12/22]). Only two previously reported STs were identified within GS1: ST29 and ST60. The remaining isolates were classified as novel STs (Figure 1).

### 3.1. Genomic Characteristics of C. concisus Isolates

Based on GC content, genome size, and ANI (Supplementary Tables S1–S3), we were able to divide the isolates into two distinct groups, corresponding to genomospecies GS1 and GS2. The results of the ANI analysis ranged from 94% to 99.8% within the same genomospecies, and around 88% to 89% when compared to other genomospecies.

### 3.2. MLST and Phylogenetic Analysis of C. concisus Isolates

MLST analysis revealed a high diversity of *C. concisus*. In each group, only one previously known sequence type (ST) was identified (ST29 in A and ST60 in B), whereas all other STs were novel (Figure 1). All the novel alleles and sequence types were deposited to the *Campylobacter* non-jejuni/coli scheme on PubMLST.

A phylogenetic tree was created by comparing the genomes of Slovenian *C. concisus* isolates with European *C. concisus* isolates available in the GenBank database (Figure 2).

### 3.3. Analysis of Plasmids in Draft C. concisus Genomes

Plasmid analysis of 40 draft *C. concisus* genomes revealed the presence of one or more plasmids in 15 *C. concisus* isolates (Table 2), mostly belonging to GS2 (11/15). There were no significant differences in the presence of plasmids between patients from Groups A and B (Table 1). In total, nine novel plasmids and six known plasmids (pADS1, pSMA1, pCCON16, pCCON31, pTJ3, and pICON) were identified (Table 2). Novel plasmids were found in both, GS1 and GS2, whereas known plasmids were only found in GS2.

### 3.4. Presence of Virulence and Resistance Genes

Analysis of the draft genomes of *C. concisus* revealed the presence of many virulence genes important for immune modulation, motility, secretory systems, or toxin production. The most prevalent virulence genes were those associated with motility, particularly genes related to chemotaxis (*cheA*, *cheV*, *cheW*, *cheY*), and flagellar structure (*flgB*, *flgC*, *flgG*, *flgH*, *flgJ*, *flgM*, *flgP*, *flgR*, *flhA*, *flhB*, *flhG*, *fliA*, *fliG*, *fliH*, *fliI*, *fliM*, *fliN*, *fliP*, *fliQ*), which were present in all isolates. In addition, two notable toxins were identified: Exo9 and Zot toxin; among 40 isolates 10 AICC (4 Group A, 6 Group B), 12 AToCC (2 Group A, 10 Group B), 3 AICC/AToCC pathotypes were found (3 Group B), with 13 without Exo9 and Zot toxin genes. Moreover, two important secretion systems, the T4SS and T6SS, were also identified in 9 and 34 isolates, respectively (Table 1). In addition, T3SS was present in all isolates.

Regarding antimicrobial resistance, no mutations associated with erythromycin resistance were found in the 23S rRNA gene (rrnA2075G). Nine isolates carried the *gyrA* T86I (C257T) mutation, which is known to confer resistance to fluoroquinolones. Two isolates harboured the *tet(O)* gene, conferring tetracycline resistance, and one isolate carried the *tet(M)* gene.

## 4. Discussion

This study used WGS analysis to investigate the genomic characteristics, phylogenetic distribution, presence of plasmids, and virulence factors in *C. concisus*, isolated from Slovenian patients with community-acquired infectious diarrhoea. In total, 59 isolates were obtained; however, only 54 were successfully revived, possibly due to the suboptimal routinely used freezing mix composition for *C. concisus* with a slightly lower concentration (10%) of cryoprotectant glycerol [32,45].

MLST analysis was consistent with previous findings that nearly all isolates represent unique STs [20]. Only two previously reported STs were identified within GS1—namely, ST29 and ST60—and the remaining isolates were classified as novel STs. GS2 contained only novel STs. This observation indicates high genetic diversity. Prior work suggests that GS2 may be better adapted to the human gut and more often associated with disease [22,44,46]. The phylogenetic analysis showed that Slovenian isolates are interdispersed with other European isolates with no discernible geographic pattern.

In *Campylobacter* spp., plasmids commonly carry antibiotic resistance determinants. In some *C. jejuni* lineages, plasmids such as pVir harbour virulence-associated genes and therefore influence their pathogenicity [27]. In addition, plasmids facilitate horizontal gene transfer. The extent and impact of plasmid-borne virulence in *C. concisus* remain less well defined. Plasmids may enhance adaptability and perhaps virulence, but their role likely varies by species and strain. In this study, plasmids were less frequently present in GS1 and more commonly found in GS2. Some isolates harboured multiple plasmids.

A number of small, high-copy-number plasmids were previously identified in *C. concisus* by Liu et al. [47], including pSma1 (~1.3 kb) with two ORFs and an estimated copy number of about 60 per cell. pSma1 and related small plasmids were detected more frequently in strains from ulcerative colitis (UC) patients that required surgical intervention due to the severity of their disease. Specifically, this plasmid was found in approximately a third of UC patients who underwent surgery, compared to a lower prevalence in Crohn’s disease patients and healthy controls [47]. Despite this association, the role of pSma1 in pathogenesis remains to be determined. pSma1 was found in two isolates from Group A belonging to GS2.

pADS1 has been reported as a plasmid in *C. concisus* in comparative genomics studies, but detailed functional characterization is lacking. References to pADS1 typically occur within broader analyses of *C. concisus* genomic diversity [47], and its role in pathogenesis remains to be clarified. Similarly, there are few available data on the pTJ3 plasmid. One isolate had both the T4SS and T6SS, whereas the other had the genes for Exo9 and Zot toxins. Both isolates had the *csep1* gene, which encodes a secreted enterotoxin homolog [10].

The pCCON16 plasmid was found in three GS2 isolates. It has been previously described as one of the most important plasmids in *C. concisus* strains, particularly in *C. concisus* reference strain ATCC BAA-145 [44]. It encodes genes that can be involved in *C. concisus* interactions with its host, contributing to its pathogenicity. In some cases, certain genes encoded on pCCON16 were also found to be integrated into the genome of different *C. concisus* strains, such as the AICC strain UNSWCD, potentially indicating genomic variation and adaptability of the plasmid [16]. The plasmid contains an origin of replication with tandem repeats, typical of plasmids, which help ensure its stable maintenance within bacterial populations [44].

The pCCON31 plasmid, found in one isolate, is one of the two plasmids (along with pCCON16) present in some *C. concisus* strains that shows lower levels of conservation and synteny when compared to other plasmids, such as the UNSWCD plasmid [16].

The pICON plasmid, found in *C. concisus* strains associated with IBD, including Crohn’s disease, was found in only two isolates. It contains a gene known as *csep1*, which encodes a secreted enterotoxin homolog. This plasmid is present in some strains of *C. concisus* from patients with active Crohn’s disease, especially those with small bowel complications. Studies have found that the *csep1* gene is more frequently found in strains from patients with active Crohn’s disease compared to healthy controls [10].

Interestingly, the previously described plasmids (pCCON16, pSma1, pADS1, pCCON31, pTJ3, and pICON) were identified in both Groups A and B, but always in GS2 isolates. This is supported by previous research because these plasmids are predominantly but not exclusively found in GS2 [47]. Two of them (pSma1 and pICON) are better studied and associated with specific diseases, pSma1 with UC and pICON with Crohn’s disease.

Plasmids in *C. concisus* contribute to virulence by carrying various virulence genes, such as *exo9*. The gene *exo9* was identified on a 30 kb plasmid found exclusively in highly invasive strains (AICC), which are associated with chronic intestinal disease. In contrast, the plasmid location of the *zot* gene (characteristic of AToCC strains) has not been conclusively confirmed in the literature [14,22]

In addition to these toxins, *C. concisus* produces virulence factors such as FlaC and CadF, secreted through the flagellar system, and some strains possess the T4SS and T6SS, enhancing their ability to adhere to and invade host cells [7,14,18,48].

The distribution of Zot and Exo9 toxin was evaluated across the *C. concisus* isolates. The Zot toxin, which disrupts tight junctions in epithelial cells and increases intestinal permeability [49,50], was identified in 15 *C. concisus* isolates: two in Group A and 13 in Group B. A statistically significant difference between the two groups was observed for the presence of Zot (*p* = 0.004; Table 1), with a higher prevalence in Group B (59.1%) compared to Group A (12.5%). In this study *zot* was primarily associated with GS1, which is consistent with previous findings [22,23]. Mahendran et al. [49] also reported a higher prevalence of the *zot* gene in strains from patients with IBD, and described several polymorphisms in the gene, suggesting functional variability among strains.

The Exo9 toxin, which is involved in disrupting host cell functions [51,52], was identified in 12 *C. concisus* isolates, mostly in Group B, but this difference was not statistically significant (*p* = 0.457; Table 1). It was associated with GS2 as previously reported [16,23].

This finding may reflect different pathogenic strategies between *C. concisus* genomospecies: one is that GS1 isolates may contribute to disease through epithelial barrier disruption due to Zot toxin, and the other is that GS2 isolates may rely on invasion and cytotoxicity because of Exo9 [14]. Deshpande et al. demonstrated that distinct *C. concisus* pathotypes induce unique global transcriptomic responses in intestinal epithelial cells, supporting the idea that genomic heterogeneity translates into functional and pathogenic diversity [14].

Three isolates (all from Group B) had genes for both Zot and Exo9 toxins, and all three belonged to GS2. The presence of both toxins in GS2 isolates is rare but has been reported previously [16,22,23]. Simultaneous presence could indicate enhanced virulence, because these isolates may combine barrier-disrupting activity with host cell cytotoxicity and invasion. Such isolates could be of particular clinical relevance and may represent a more pathogenic subtype within the *C. concisus* population. The patients with these three isolates containing both toxins also harbored well-established enteric pathogens (*Salmonella enteritidis*, *Shigella flexneri* type 6 and Rotavirus), which were presumed to be the primary etiological agents of their community acquired infectious diarrhoea. Most published work on Zot and Exo9 toxins positive *C. concisus* isolates relates to chronic intestinal disease (IBD, microscopic colitis) rather than acute infectious gastroenteritis [7,8,22].

In addition to these toxins, the presence of secretory systems was analysed [22]. The T3SS was detected in all isolates, and T5SS was not detected. The T6SS is a more recently discovered secretion system that plays a dual role in both interbacterial competition and host–pathogen interactions [53,54]. The T6SS is also crucial for virulence through its ability to inject effector proteins into host cells. These effectors can induce host cell death or disrupt normal cellular functions, further promoting bacterial survival and dissemination. Pathogens such as *Pseudomonas aeruginosa* and *Vibrio cholerae* utilize the T6SS to establish infections by targeting host immune cells or manipulating local tissue structures, thus exacerbating inflammation and promoting tissue damage. This ability to eliminate competing microbiota enhances the survival and establishment of pathogens in the host environment, which is critical for the persistence of infection [53,55,56]. Our isolates with the T6SS belonged to both Groups A and B, and no statistically significant difference was observed between the two groups (*p* = 0.635; Table 1).

Importantly, 8/40 isolates with the T4SS also carried the T6SS, reinforcing the concept of combined secretion systems in particular isolates [22], and no statistically significant difference was observed between Groups A and B (*p* = 0.350, Table 1). The T4SS is known to mediate DNA and protein transfer and to play a major role in bacterial adaptation and virulence [57]. The presence of the T4SS and T6SS supports the idea that certain *Campylobacter* isolates possess complex virulence strategies combining interbacterial antagonism with DNA/protein transfer capabilities.

This study also analysed the presence of antimicrobial resistance genes in *C. concisus* isolates. No macrolide resistance genes were identified in any of the isolates. Kaakoush et al. reported a low level of resistance to erythromycin (≈2.5%) among a population of 73 isolates [7]. Mutations in the quinolone resistance-determining region (QRDR) of the *gyrA* gene were detected in nine isolates, such as the C257T substitution leading to the Thr-86-Ile amino acid change, which is the predominant fluoroquinolone resistance determinant [58]. Six out of nine were from GS2, indicating the presence of fluoroquinolone resistance in a subset of the population. Tetracycline resistance was confirmed in three isolates. Two of these harboured the *tet(O)* gene, which encodes a ribosomal protection protein, and the third carried the *tet(M)* gene, also associated with ribosomal protection and resistance to tetracyclines. The detection of these genes, particularly *tet(O)* and *tet(M)*, raises the possibility of horizontal gene transfer events occurring within the intestinal microbiota. Overall, our results indicate that although a proportion of *C. concisus* isolates have acquired resistance to ciprofloxacin and tetracycline, the species remains largely susceptible to macrolides. Nevertheless, the presence of resistance genes underscores the importance of continued surveillance and further investigation into the resistome of *C. concisus*.

Our study provided insight into the genotypic variability of clinical *C. concisus* isolates from patients with community-acquired infectious diarrhoea in Slovenia. We found that the genetic heterogeneity of clinical *C. concisus* isolates is high. The gene profiles for virulence factors are not directly linked to the determination of ST but are associated with GS. By identifying plasmids and genes associated with resistance and virulence factors, we gained a better understanding of *C. concisus* virulence.

## 5. Conclusions

This study provides the first in-depth whole-genome sequencing analysis of *C. concisus* isolates from Slovenian patients with infectious diarrhoea. *C. concisus* isolates from patients, in whom no other gastrointestinal pathogens were detected, were predominantly assigned to genomospecies GS2, characterized by larger genomes, higher GC content, and a greater number of coding regions. These isolates showed a higher prevalence of plasmids associated with virulence, including the *exo9* toxin gene and the conjugative T4SS and T6SS, suggesting enhanced pathogenic potential and adaptability. In contrast, *C. concisus* isolates from patients, in whom other gastrointestinal pathogens were detected simultaneously, were mainly from genomospecies GS1, and they had smaller genomes and more frequent presence of the *zot* toxin gene. The majority of isolates harboured the T6SS, emphasizing its fundamental role in *C. concisus* biology. This genomic insight advances the understanding of *C. concisus* pathogenicity.

## Figures and Tables

**Table 1:** Association between genomospecies/virulence genes and Group A and B in *Campylobacter concisus* isolates.

Variable	Category	Frequencies (%) A (*n* = 16)	Frequencies (%) B (*n* = 22)	*p*-Value
Genomospecies	GS1	7 (43.8%)	12 (54.5%)	0.511
	GS2	9 (56.3%)	10 (45.5%)	
Plasmids	Present	7 (43.8%)	8 (36.4%)	0.646
	Absent	9 (56.3%)	14 (63.6%)	
Zot	Present	2 (12.5%)	13 (59.1%)	**0.004**
	Absent	14 (87.5%)	9 (40.9%)	
Exo9	Present	4 (25.0%)	8 (36.4%)	0.457
	Absent	12 (75.0%)	14 (63.6%)	
T6SS	Present	14 (87.5%)	18 (81.8%)	0.635
	Absent	2 (12.5%)	4 (18.2%)	
T4SS	Present	5 (31.3%)	4 (18.2%)	0.350
	Absent	11 (68.8%)	18 (81.8%)	

Notes: bold *p*-values (<0.05) indicate statistical significance. T6SS = Type VI secretion system; T4SS = Type IV secretion system; Zot = Zonula occludens toxin; Exo9 = Exotoxin9/DnaI.

**Table 2:** Characterization of detected plasmids in *C. concisus* isolates.

Isolate	Blast Result	Length (bp)	GS	Group
Cco_SLO_14	pADS1	3982	2	A
Cco_SLO_15	pCCON31	35,000	2	A
Cco_SLO_18	novel plasmid	7690	1	B
Cco_SLO_19	novel plasmid	28,981	1	B
Cco_SLO_22	novel plasmid	9766	2	A
	pSMA1	1423	2	A
	pCCON16	19,000	2	A
Cco_SLO_23	novel plasmid	2631	2	A
	pSma1	1401	2	A
Cco_SLO_27	novel plasmid	28,981	1	B
Cco_SLO_29	pTJ3	3921	2	B
	pICON	117,000	2	B
Cco_SLO_31	pCCON16	13,400	2	B
Cco_SLO_32	novel plasmid	1475	2	A
Cco_SLO_35	novel plasmid	3914	2	B
	pICON	118,300	2	B
Cco_SLO_36	pADS1	3703	2	A
	pCCON16	15,200	2	A
Cco_SLO_37	novel plasmid	28,931	1	B
Cco_SLO_3	pTJ3	4137	2	B
Cco_SLO_9	novel plasmid	25,761	2	A
	pCCON16	15,000	2	A

## Data Availability

The genomic data associated with this study can be found under the BioProject accession number PRJEB98669.

## Supplementary Materials

The following supporting information can be downloaded at: https://www.mdpi.com/article/10.3390/microorganisms14010087/s1, Table S1. Genome information data; Table S2. Pangenome information; Table S3. Average nucleotide identity matrix.

## Abbreviations

The following abbreviations are used in this manuscript:

GS1	Genomospecies 1
GS2	Genomospecies 2
AICC	Adherent-invasive *C. concisus*
AToCC	Adherent-toxigenic *C. concisus*
T6SS	Type VI secretion system
T4SS	Type IV secretion system
Zot	Zonula occludens toxin
Exo9	Exotoxin9/DnaI toxin
WGS	Whole-genome sequencing
MLST	Multilocus sequence typing
