# Supplementary material for: Genetic Diversity of Campylobacter concisus Isolates from Slovenian Patients with Infectious Diarrhoea
Source: Microorganisms. 2025 Dec 31;14(1):87. doi: 10.3390/microorganisms14010087 (PMC12844130; doi:10.3390/microorganisms14010087)
Supplement: Supplementary file 1 [file microorganisms-14-00087-s001.zip › Table S1.pdf]

Table S1. Genome information data.

Sample	Genome size (bp)	GC content (%)	Coding regions (n)	Genomospecies
Cco_SLO_1	1792986	37,55	1752	GS1
Cco_SLO_10	1889766	37,52	1867	GS1
Cco_SLO_11	1888875	37,48	1909	GS1
Cco_SLO_12	1864136	37,5	1811	GS1
Cco_SLO_13	1896846	37,6	1904	GS1
Cco_SLO_16	1783612	37,63	1772	GS1
Cco_SLO_18	1887827	37,32	1871	GS1
Cco_SLO_19	1926758	37,51	1933	GS1
Cco_SLO_2	1860197	37,49	1858	GS1
Cco_SLO_25	1885146	37,6	1896	GS1
Cco_SLO_27	1923627	37,21	1916	GS1
Cco_SLO_30	1891482	37,53	1857	GS1
Cco_SLO_33	1798677	37,6	1783	GS1
Cco_SLO_34	1902300	37,45	1901	GS1
Cco_SLO_37	1828320	37,43	1812	GS1
Cco_SLO_38	1842697	37,38	1809	GS1
Cco_SLO_4	1911862	37,43	1927	GS1
Cco_SLO_40	1824031	37,37	1792	GS1
Cco_SLO_5	1822138	37,63	1803	GS1
Cco_SLO_6	1808461	37,69	1780	GS1
Cco_SLO_14	2087127	39,32	2015	GS2
Cco_SLO_15	2007313	39,31	1960	GS2
Cco_SLO_17	1933084	39,36	1883	GS2
Cco_SLO_20	1999500	39,59	1914	GS2
Cco_SLO_21	2043996	39,31	2004	GS2
Cco_SLO_22	2000954	39,48	1949	GS2
Cco_SLO_23	1979944	39,57	1901	GS2
Cco_SLO_24	2019838	39,5	1960	GS2
Cco_SLO_26	2001604	39,38	1945	GS2
Cco_SLO_28	1883403	39,61	1834	GS2
Cco_SLO_29	2169023	39,04	2135	GS2
Cco_SLO_3	1943106	39,56	1916	GS2
Cco_SLO_31	2087084	39,11	2069	GS2
Cco_SLO_32	2010080	39,42	1943	GS2
Cco_SLO_35	2150888	39,02	2149	GS2
Cco_SLO_36	1904564	39,61	1897	GS2
Cco_SLO_39	2051105	39,21	2027	GS2
Cco_SLO_7	1979861	39,56	1936	GS2
Cco_SLO_8	2057769	39,25	1975	GS2
Cco_SLO_9	2036198	39,47	1990	GS2
