# Supplementary material for: Genetic Diversity of Campylobacter concisus Isolates from Slovenian Patients with Infectious Diarrhoea
Source: Microorganisms. 2025 Dec 31;14(1):87. doi: 10.3390/microorganisms14010087 (PMC12844130; doi:10.3390/microorganisms14010087)
Supplement: Supplementary file 1 [file microorganisms-14-00087-s001.zip › Table S2.pdf]

**Table S2.** Pangenome information.

| **Pangenome** | **Genomospecies 1 (n=20)** | **n** |
|---|---|---|
| Core genes | (99% <= strains <= 100%) | 1039 |
| Soft core genes | (95% <= strains < 99%) | 174 |
| Shell genes | (15% <= strains < 95%) | 1210 |
| Cloud genes | (0% <= strains < 15%) | 3158 |
| Total genes | (0% <= strains <= 100%) | 5581 |
| **Pangenome** | **Genomospecies 2 (n=20)** | **n** |
| Core genes | (99% <= strains <= 100%) | 1163 |
| Soft core genes | (95% <= strains < 99%) | 96 |
| Shell genes | (15% <= strains < 95%) | 1205 |
| Cloud genes | (0% <= strains < 15%) | 3967 |
| Total genes | (0% <= strains <= 100%) | 6431 |
| **Pangenome** | **All samples (n=40)** | **n** |
| Core genes | (99% <= strains <= 100%) | 618 |
| Soft core genes | (95% <= strains < 99%) | 57 |
| Shell genes | (15% <= strains < 95%) | 2248 |
| Cloud genes | (0% <= strains < 15%) | 7490 |
| Total genes | (0% <= strains <= 100%) | 10413 |
