# Supplementary material for: Genetic Diversity of Campylobacter concisus Isolates from Slovenian Patients with Infectious Diarrhoea
Source: Microorganisms. 2025 Dec 31;14(1):87. doi: 10.3390/microorganisms14010087 (PMC12844130; doi:10.3390/microorganisms14010087)
Supplement: Supplementary file 1 [file microorganisms-14-00087-s001.zip › Table S3.pdf]

ANI Matrix	Cco_SLO_21	Cco_SLO_39	Cco_SLO_28	Cco_SLO_17	Cco_SLO_36	Cco_SLO_9	Cco_SLO_14	Cco_SLO_22	Cco_SLO_8	Cco_SLO_20	Cco_SLO_23	Cco_SLO_26	Cco_SLO_29	Cco_SLO_32	Cco_SLO_15	Cco_SLO_24	Cco_SLO_7	Cco_SLO_31	GCF_003048685.2	Cco_SLO_3	Cco_SLO_35	Cco_SLO_34	Cco_SLO_4	Cco_SLO_11	Cco_SLO_10	Cco_SLO_12	Cco_SLO_30	GCF_001298465.1	Cco_SLO_5	Cco_SLO_38	Cco_SLO_40	Cco_SLO_25	Cco_SLO_27	Cco_SLO_6	Cco_SLO_1	Cco_SLO_2	Cco_SLO_33	Cco_SLO_18	Cco_SLO_13	Cco_SLO_37	Cco_SLO_19	Cco_SLO_16
**Cco_SLO_21**	100	99,0	96,0	95,8	94,9	94,7	94,7	94,6	94,9	94,8	94,8	94,8	94,7	94,9	94,7	94,8	94,8	94,2	94,0	94,3	94,0	88,3	88,5	88,4	88,9	88,6	88,7	88,7	88,7	88,4	88,4	88,8	88,6	88,6	88,7	88,5	88,7	88,5	88,8	88,5	88,5	88,8
**Cco_SLO_39**	99,0	100	96,0	95,8	95,0	94,7	94,7	94,6	94,9	94,8	94,7	94,9	94,8	94,9	94,8	94,9	94,8	94,2	94,1	94,2	94,0	88,4	88,5	88,4	88,8	88,6	88,6	88,6	88,6	88,4	88,4	88,9	88,7	88,6	88,8	88,6	88,5	88,5	88,8	88,5	88,5	88,8
**Cco_SLO_28**	96,0	96,0	100	96,0	95,0	94,8	94,7	94,7	95,1	95,0	94,9	94,9	94,8	95,0	94,9	95,0	94,8	94,2	94,2	94,3	94,1	88,5	88,5	88,4	88,9	88,6	88,7	88,9	88,7	88,6	88,6	89,0	88,6	88,8	88,7	88,6	88,6	88,7	88,8	88,8	88,5	88,8
**Cco_SLO_17**	95,8	95,8	96,0	100	95,0	94,8	94,8	94,5	94,9	94,9	94,8	94,8	94,8	94,9	94,6	95,0	94,9	94,3	94,2	94,5	94,1	88,4	88,5	88,4	88,8	88,6	88,6	88,7	88,7	88,4	88,4	88,8	88,6	88,6	88,7	88,5	88,4	88,5	88,7	88,5	88,4	88,9
**Cco_SLO_36**	94,9	95,0	95,0	95,0	100	95,0	95,0	94,9	95,0	95,0	95,0	95,0	95,0	95,0	94,9	95,3	94,9	94,6	94,4	94,6	94,6	88,5	88,5	88,4	88,6	88,6	88,7	88,8	88,8	88,4	88,5	89,0	88,8	88,8	88,8	88,7	88,7	88,7	88,8	88,5	88,8	88,8
**Cco_SLO_9**	94,7	94,7	94,8	94,8	95,0	100	97,5	95,2	95,2	95,2	95,2	95,1	95,3	95,5	95,4	95,3	95,4	94,7	94,8	95,0	94,5	88,2	88,2	88,1	88,4	88,4	88,4	88,4	88,6	88,2	88,3	88,8	88,6	88,5	88,5	88,3	88,5	88,6	88,5	88,5	88,4	88,8
**Cco_SLO_14**	94,7	94,7	94,7	94,8	95,0	97,5	100	95,2	95,1	95,0	95,2	95,1	95,3	95,6	95,4	95,3	95,2	94,8	94,8	95,0	94,6	88,5	88,3	88,3	88,4	88,4	88,4	88,6	88,6	88,3	88,3	88,8	88,6	88,6	88,5	88,3	88,4	88,7	88,4	88,4	88,4	88,6
**Cco_SLO_22**	94,6	94,6	94,7	94,5	94,9	95,2	95,2	100	95,1	95,1	95,1	95,1	95,2	95,3	95,2	95,2	95,1	94,8	94,8	94,9	94,7	88,2	88,2	88,1	88,5	88,4	88,4	88,3	88,4	88,3	88,3	88,6	88,6	88,5	88,5	88,3	88,4	88,6	88,5	88,3	88,5	88,5
**Cco_SLO_8**	94,9	94,9	95,1	94,9	95,0	95,2	95,1	95,1	100	95,4	95,3	95,3	95,2	95,4	95,2	95,4	95,3	94,5	94,6	94,8	94,5	88,2	88,2	88,2	88,5	88,4	88,5	88,5	88,6	88,1	88,3	88,8	88,4	88,6	88,6	88,4	88,4	88,4	88,4	88,4	88,3	88,6
**Cco_SLO_20**	94,8	94,8	95,0	94,9	95,0	95,2	95,0	95,1	95,4	100	95,2	95,2	95,2	95,2	95,1	95,3	95,2	94,6	94,6	94,8	94,5	88,4	88,3	88,3	88,5	88,4	88,5	88,6	88,5	88,2	88,2	88,7	88,4	88,5	88,6	88,5	88,5	88,6	88,5	88,5	88,4	88,6
**Cco_SLO_23**	94,8	94,7	94,9	94,8	95,0	95,2	95,2	95,1	95,3	95,2	100	95,4	95,2	95,4	95,3	95,3	95,2	94,6	94,8	94,8	94,6	88,3	88,2	88,1	88,5	88,4	88,5	88,6	88,6	88,2	88,3	88,5	88,2	88,5	88,5	88,4	88,3	88,4	88,6	88,5	88,4	88,5
**Cco_SLO_26**	94,8	94,9	94,9	94,8	95,0	95,1	95,1	95,1	95,3	95,2	95,4	100	95,2	95,3	95,3	95,3	95,2	94,6	94,7	94,8	94,6	88,2	88,3	88,3	88,6	88,5	88,4	88,8	88,8	88,3	88,4	88,8	88,4	88,6	88,6	88,5	88,4	88,5	88,6	88,5	88,4	88,7
**Cco_SLO_29**	94,7	94,8	94,8	94,8	95,0	95,3	95,3	95,2	95,2	95,2	95,2	95,2	100	95,4	95,5	95,3	95,4	94,6	94,7	94,9	94,7	88,3	88,3	88,2	88,5	88,5	88,5	88,6	88,5	88,3	88,3	88,7	88,4	88,6	88,5	88,4	88,5	88,4	88,6	88,4	88,4	88,8
**Cco_SLO_32**	94,9	94,9	95,0	94,9	95,0	95,5	95,6	95,3	95,4	95,2	95,4	95,3	95,4	100	95,6	95,5	95,4	94,8	94,7	94,9	94,6	88,4	88,3	88,2	88,5	88,4	88,5	88,5	88,7	88,2	88,2	88,8	88,9	88,7	88,7	88,4	88,5	88,7	88,6	88,4	88,4	88,6
**Cco_SLO_15**	94,7	94,8	94,9	94,6	94,9	95,4	95,4	95,2	95,2	95,1	95,3	95,3	95,5	95,6	100	95,3	95,5	94,8	94,7	95,0	94,5	88,3	88,2	88,2	88,6	88,5	88,4	88,7	88,7	88,2	88,3	88,7	88,5	88,5	88,6	88,2	88,3	88,6	88,5	88,2	88,4	88,8
**Cco_SLO_24**	94,8	94,9	95,0	95,0	95,3	95,3	95,3	95,2	95,4	95,3	95,3	95,3	95,3	95,5	95,3	100	95,5	94,6	94,7	94,8	94,5	88,3	88,4	88,3	88,5	88,4	88,5	88,6	88,7	88,3	88,3	88,8	88,5	88,5	88,5	88,4	88,5	88,5	88,7	88,4	88,4	88,8
**Cco_SLO_7**	94,8	94,8	94,8	94,9	94,9	95,4	95,2	95,1	95,3	95,2	95,2	95,2	95,4	95,4	95,5	95,5	100	94,6	94,7	95,0	94,6	88,4	88,3	88,3	88,6	88,5	88,5	88,6	88,7	88,4	88,5	88,7	88,5	88,6	88,6	88,5	88,4	88,5	88,4	88,5	88,4	88,5
**Cco_SLO_31**	94,2	94,2	94,2	94,3	94,6	94,7	94,8	94,8	94,5	94,6	94,6	94,6	94,6	94,8	94,8	94,6	94,6	100	94,6	94,8	94,6	88,2	88,4	88,2	88,4	88,2	88,3	88,5	88,4	88,2	88,0	88,7	88,7	88,4	88,4	88,4	88,2	88,6	88,5	88,5	88,3	88,7
**GCF_003048685.2**	94,0	94,1	94,2	94,2	94,4	94,8	94,8	94,8	94,6	94,6	94,8	94,7	94,7	94,7	94,7	94,7	94,7	94,6	100	94,9	94,7	88,2	88,2	88,1	88,3	88,2	88,2	88,7	88,6	88,1	88,1	88,5	88,3	88,3	88,3	88,2	88,2	88,2	88,3	88,5	88,1	88,5
**Cco_SLO_3**	94,3	94,2	94,3	94,5	94,6	95,0	95,0	94,9	94,8	94,8	94,8	94,8	94,9	94,9	95,0	94,8	95,0	94,8	94,9	100	95,0	88,3	88,2	88,2	88,5	88,4	88,5	88,8	88,7	88,2	88,3	88,7	88,3	88,5	88,5	88,3	88,4	88,3	88,5	88,4	88,2	88,7
**Cco_SLO_35**	94,0	94,0	94,1	94,1	94,6	94,5	94,6	94,7	94,5	94,5	94,6	94,6	94,7	94,6	94,5	94,5	94,6	94,6	94,7	95,0	100	88,3	88,3	88,3	88,3	88,4	88,4	88,6	88,7	88,2	88,1	88,7	88,5	88,4	88,4	88,4	88,4	88,4	88,5	88,4	88,4	88,8
**Cco_SLO_34**	88,3	88,4	88,5	88,4	88,5	88,2	88,5	88,2	88,2	88,4	88,3	88,2	88,3	88,4	88,3	88,3	88,4	88,2	88,2	88,3	88,3	100	98,4	98,5	94,3	94,4	94,5	94,3	94,3	94,5	94,5	94,5	94,7	94,4	94,5	94,5	94,5	94,6	94,5	94,3	94,2	94,4
**Cco_SLO_4**	88,5	88,5	88,5	88,5	88,5	88,2	88,3	88,2	88,2	88,3	88,2	88,3	88,3	88,3	88,2	88,4	88,3	88,4	88,2	88,2	88,3	98,4	100	98,9	94,4	94,5	94,4	94,3	94,4	94,5	94,5	94,4	94,7	94,6	94,5	94,5	94,5	94,6	94,4	94,4	94,3	94,5
**Cco_SLO_11**	88,4	88,4	88,4	88,4	88,4	88,1	88,3	88,1	88,2	88,3	88,1	88,3	88,2	88,2	88,2	88,3	88,3	88,2	88,1	88,2	88,3	98,5	98,9	100	94,3	94,4	94,3	94,2	94,3	94,4	94,5	94,4	94,7	94,4	94,5	94,5	94,6	94,6	94,4	94,3	94,2	94,5
**Cco_SLO_10**	88,9	88,8	88,9	88,8	88,6	88,4	88,4	88,5	88,5	88,5	88,5	88,6	88,5	88,5	88,6	88,5	88,6	88,4	88,3	88,5	88,3	94,3	94,4	94,3	100	99,1	99,0	94,3	94,2	94,9	95,0	94,5	94,8	94,9	95,0	95,0	95,2	95,5	95,6	94,3	94,5	94,5
**Cco_SLO_12**	88,6	88,6	88,6	88,6	88,6	88,4	88,4	88,4	88,4	88,4	88,4	88,5	88,5	88,4	88,5	88,4	88,5	88,2	88,2	88,4	88,4	94,4	94,5	94,4	99,1	100	99,8	94,2	94,3	94,9	95,0	94,7	94,8	94,9	95,0	95,0	95,2	95,5	95,6	94,3	94,5	94,4
**Cco_SLO_30**	88,7	88,6	88,7	88,6	88,7	88,4	88,4	88,4	88,5	88,5	88,5	88,4	88,5	88,5	88,4	88,5	88,5	88,3	88,2	88,5	88,4	94,5	94,4	94,3	99,0	99,8	100	94,2	94,2	94,9	95,0	94,6	94,7	94,9	95,0	94,9	95,2	95,5	95,6	94,3	94,5	94,4
**GCF_001298465.1**	88,7	88,6	88,9	88,7	88,8	88,4	88,6	88,3	88,5	88,6	88,6	88,8	88,6	88,5	88,7	88,6	88,6	88,5	88,7	88,8	88,6	94,3	94,3	94,2	94,3	94,2	94,2	100	98,7	94,2	94,2	94,3	94,3	94,2	94,3	94,2	94,3	94,3	94,4	94,4	94,2	94,4
**Cco_SLO_5**	88,7	88,6	88,7	88,7	88,8	88,6	88,6	88,4	88,6	88,5	88,6	88,8	88,5	88,7	88,7	88,7	88,7	88,4	88,6	88,7	88,7	94,3	94,4	94,3	94,2	94,3	94,2	98,7	100	94,2	94,2	94,4	94,4	94,1	94,3	94,2	94,3	94,3	94,3	94,4	94,1	94,2
**Cco_SLO_38**	88,4	88,4	88,6	88,4	88,4	88,2	88,3	88,3	88,1	88,2	88,2	88,3	88,3	88,2	88,2	88,3	88,4	88,2	88,1	88,2	88,2	94,5	94,5	94,4	94,9	94,9	94,9	94,2	94,2	100	99,7	94,5	94,7	94,8	94,9	94,8	94,9	95,1	95,0	94,2	94,3	94,6
**Cco_SLO_40**	88,4	88,4	88,6	88,4	88,5	88,3	88,3	88,3	88,3	88,2	88,3	88,4	88,3	88,2	88,3	88,3	88,5	88,0	88,1	88,3	88,1	94,5	94,5	94,5	95,0	95,0	95,0	94,2	94,2	99,7	100	94,6	94,7	94,9	95,0	94,9	94,9	95,2	95,1	94,2	94,3	94,6
**Cco_SLO_25**	88,8	88,9	89,0	88,8	89,0	88,8	88,8	88,6	88,8	88,7	88,5	88,8	88,7	88,8	88,7	88,8	88,7	88,7	88,5	88,7	88,7	94,5	94,4	94,4	94,5	94,7	94,6	94,3	94,4	94,5	94,6	100	94,7	94,7	94,7	94,6	94,6	94,8	94,6	94,4	94,4	94,5
**Cco_SLO_27**	88,6	88,7	88,6	88,6	88,8	88,6	88,6	88,6	88,4	88,4	88,2	88,4	88,4	88,9	88,5	88,5	88,5	88,7	88,3	88,3	88,5	94,7	94,7	94,7	94,8	94,8	94,7	94,3	94,4	94,7	94,7	94,7	100	94,7	94,8	94,8	94,7	94,9	94,8	94,4	94,5	94,6
**Cco_SLO_6**	88,6	88,6	88,8	88,6	88,8	88,5	88,6	88,5	88,6	88,5	88,5	88,6	88,6	88,7	88,5	88,5	88,6	88,4	88,3	88,5	88,4	94,4	94,6	94,4	94,9	94,9	94,9	94,2	94,1	94,8	94,9	94,7	94,7	100	95,0	94,8	94,7	95,0	95,0	94,2	94,4	94,5
**Cco_SLO_1**	88,7	88,8	88,7	88,7	88,8	88,5	88,5	88,5	88,6	88,6	88,5	88,6	88,5	88,7	88,6	88,5	88,6	88,4	88,3	88,5	88,4	94,5	94,5	94,5	95,0	95,0	95,0	94,3	94,3	94,9	95,0	94,7	94,8	95,0	100	94,9	94,9	95,1	95,1	94,3	94,5	94,5
**Cco_SLO_2**	88,5	88,6	88,6	88,5	88,7	88,3	88,3	88,3	88,4	88,5	88,4	88,5	88,4	88,4	88,2	88,4	88,5	88,4	88,2	88,3	88,4	94,5	94,5	94,5	95,0	95,0	94,9	94,2	94,2	94,8	94,9	94,6	94,8	94,8	94,9	100	94,9	95,2	95,0	94,3	94,4	94,6
**Cco_SLO_33**	88,7	88,5	88,6	88,4	88,7	88,5	88,4	88,4	88,4	88,5	88,3	88,4	88,5	88,5	88,3	88,5	88,4	88,2	88,2	88,4	88,4	94,5	94,5	94,6	95,2	95,2	95,2	94,3	94,3	94,9	94,9	94,6	94,7	94,7	94,9	94,9	100	95,3	95,2	94,3	94,5	94,5
**Cco_SLO_18**	88,5	88,5	88,7	88,5	88,7	88,6	88,7	88,6	88,4	88,6	88,4	88,5	88,4	88,7	88,6	88,5	88,5	88,6	88,2	88,3	88,4	94,6	94,6	94,6	95,5	95,5	95,5	94,3	94,3	95,1	95,2	94,8	94,9	95,0	95,1	95,2	95,3	100	95,6	94,4	94,6	94,7
**Cco_SLO_13**	88,8	88,8	88,8	88,7	88,8	88,5	88,4	88,5	88,4	88,5	88,6	88,6	88,6	88,6	88,5	88,7	88,4	88,5	88,3	88,5	88,5	94,5	94,4	94,4	95,6	95,6	95,6	94,4	94,3	95,0	95,1	94,6	94,8	95,0	95,1	95,0	95,2	95,6	100	94,4	94,6	94,6
**Cco_SLO_37**	88,5	88,5	88,8	88,5	88,5	88,5	88,4	88,3	88,4	88,5	88,5	88,5	88,4	88,4	88,2	88,4	88,5	88,5	88,5	88,4	88,4	94,3	94,4	94,3	94,3	94,3	94,3	94,4	94,4	94,2	94,2	94,4	94,4	94,2	94,3	94,3	94,3	94,4	94,4	100	94,4	94,5
**Cco_SLO_19**	88,5	88,5	88,5	88,4	88,8	88,4	88,4	88,5	88,3	88,4	88,4	88,4	88,4	88,4	88,4	88,4	88,4	88,3	88,1	88,2	88,4	94,2	94,3	94,2	94,5	94,5	94,5	94,2	94,1	94,3	94,3	94,4	94,5	94,4	94,5	94,4	94,5	94,6	94,6	94,4	100	94,5
**Cco_SLO_16**	88,8	88,8	88,8	88,9	88,8	88,8	88,6	88,5	88,6	88,6	88,5	88,7	88,8	88,6	88,8	88,8	88,5	88,7	88,5	88,7	88,8	94,4	94,5	94,5	94,5	94,4	94,4	94,4	94,2	94,6	94,6	94,5	94,6	94,5	94,5	94,6	94,5	94,7	94,6	94,5	94,5	100
